# Identifying Informal Settlements Using Contourlet Assisted Deep Learning

**DOI:** 10.3390/s20092733

**Published:** 2020-05-11

**Authors:** Rizwan Ahmed Ansari, Rakesh Malhotra, Krishna Mohan Buddhiraju

**Affiliations:** 1 Department of Environmental, Earth and Geospatial Sciences, North Carolina Central University, Durham, NC 27707, USA; rmalhotra@nccu.edu; 2 Centre of Studies in Resources Engineering, Indian Institute of Technology Bombay, Mumbai 400076, India; bkmohan@csre.iitb.ac.in

**Keywords:** remote sensing, informal settlements, multiresolution, deep learning, contourlet transform, semantic segmentation

## Abstract

As the global urban population grows due to the influx of migrants from rural areas, many cities in developing countries face the emergence and proliferation of unplanned and informal settlements. However, even though the rise of unplanned development influences planning and management of residential land-use, reliable and detailed information about these areas is often scarce. While formal settlements in urban areas are easily mapped due to their distinct features, this does not hold true for informal settlements because of their microstructure, instability, and variability of shape and texture. Therefore, detecting and mapping these areas remains a challenging task. This research will contribute to the development of tools to identify such informal built-up areas by using an integrated approach of multiscale deep learning. The authors propose a composite architecture for semantic segmentation using the U-net architecture aided by information obtained from a multiscale contourlet transform. This work also analyzes the effects of wavelet and contourlet decompositions in the U-net architecture. The performance was evaluated in terms of precision, recall, F-score, mean intersection over union, and overall accuracy. It was found that the proposed method has better class-discriminating power as compared to existing methods and has an overall classification accuracy of 94.9–95.7%.

## 1. Introduction

Due to rapid urbanization and population migration, many cities in developing countries such as India have large areas of unplanned development interspaced with planned areas. A forecast of the United Nations (UN) estimated that the population of India will be about 1.44 billion in 2024 and will surpass 1.66 billion around 2050 [1]. This increase, coupled with migration from rural areas to urban centres, will lead to the growth of both informal and formal urban settlements that include low-, medium-, and upper-class housing and commercial developments. Urbanization is often not accompanied by adequate development of infrastructure, including housing, sanitation, and transportation corridors. This lack of planning when coupled with the large share of informal low-paid employment results in the growth of informal settlements in densely populated urban areas [2]. The identification and mapping of informal settlements plays an important role in many applications, such as urban analysis, updating geographical databases, land cover change assessment, disaster management, and extraction of thematic information. However, connecting reliable information for these areas with accurate detection and classification of informal settlements using remote sensing remains a challenge. Demarcating and differentiating urban structures is difficult due to the nature and intermingling of these classes as opposed to the cleaner separation of standard land cover classes in planned areas with clearer differentiations in classes. This requires the extraction and analysis of textural and spatial features and dissimilar vegetation classes, along with urban structures lacking unique and easily distinguishable spectral signatures [3]. In other words, different urban classes may present similar spectral values that make it more challenging to accurately classify pixels when identifying informal settlements.

## 2. Related Work

Several studies have addressed this problem by focusing on the physical characteristics of informally settled areas when analyzing them using remotely sensed images [4,5,6]. Many of these methods use object-based [7,8] and pixel-based classification techniques [9,10]. In pixel-based methods, it is important to understand and infer objects and their spatial relationships in an image [11,12,13]. Spatial information extraction techniques such as those based on the grey level co-occurrence matrix (GLCM) have been employed to extract the underlying texture in the image to achieve more accurate classification, while texture-feature-based classification techniques were also explored in a few applications [14] in combination with support vector machines to improve performance [15,16]. 

Multiresolution analysis (MRA) techniques have been used for textural analysis and semantic segmentation [17,18]. An MRA method decomposes an image into low- and high-frequency subbands at various scales for analysis and interpretation. Wavelet-based multiresolution features were utilized for semantic segmentation of remotely sensed images to capture multiscale characteristics of different objects [19,20]. Although the two dimensional orthogonal wavelet-based MRA captures linear directional information [21], it is generally used in many applications, including image segmentation [22,23]. A range of other basis functions have been used to extend traditional wavelets, which capture non-linear discontinuities at different scales and aspect ratios to better represent an edge. A conceptual extension of wavelet-based MRA is a contourlet transform [24], which aims to overcome the representational constraints of wavelets. The contourlet-based texture features are used for slum identification in remotely sensed images [20]. The contourlet-based segmentation provides improved performance over the wavelet-based method in [20]. In this work, the utility of contourlet subbands in a deep learning framework is investigated as an extension to the previous work on MRA-based segmentation [20] for the same problem.

In the past, continued progress has been witnessed using deep learning, which has achieved state-of-the-art performance in image and information processing domains, including remote sensing applications [25,26,27,28]. A variety of neural network architectures have been studied, including convolutional neural networks and their variants [29,30,31,32]. These methods provide better results and show great potential in applying deep learning techniques to analyze remote sensing tasks. Deep neural networks have been used in remote sensing for classification [33,34] and urban analysis [35,36]. Various methods utilizing wavelet-based features in neural networks have also been explored to capitalize on multiscale features of wavelets in the computer vision domain [37,38,39]. Multiscale convolutional neural networks have also been used for classification [40,41]. Fully convolutional networks have shown improved performance for classification [42,43,44]. One such network was able to detect different classes and identify their shapes, such as built-up areas, road curvature, and vegetation boundaries. However, it was not capable of detecting small objects and classes with many internal boundaries, because the boundaries of these objects may be blurred or not properly oriented, meaning the results are comparatively degraded [45].

Several studies have aimed to improve the performance of segmentation using deep neural network structures by incorporating high-frequency data, which manifest the detailed information of an image [46,47,48,49,50]. However, studies have also shown that it is very difficult to train a deep architecture due to problems such as vanishing gradients. To overcome this problem, a U-net-based architecture that concatenates features at various scales is proposed in [51]. This architecture combines coarser and detailed semantic information at different scales to achieve better performance in biomedical image segmentation.

The architecture in [52] has the ability to work with small training data, yet provides improved results. The U-net is a convolutional network architecture without fully connected layers, which are found in most of the neural networks. It has an encoder and a decoder. The encoder consists of down-samplers, convolutional units, and max-pooling layers. The U-net architecture is designed as an improvement of the fully connected neural network (FCN) specifically for semantic segmentation [53]. The architectural advantage of the U-net over FCNs is its symmetricity and bottleneck layers, which combine the information from encoders and decoders by the process of concatenation, whereas these are summed in FCNs. Additionally, while performing the down-sampling in FCN, the receptive field may reduce the resolution, which in turn results in loss of detailed information [52]. Unlike a general convolutional neural network, U-net does not include a fully connected layer, meaning it does not require large datasets. In this study, the U-net is modified to combine the directional subbands of contourlet transforms for semantic segmentation of informal settlement areas in remotely sensed images.

Neural networks utilizing multiscale contourlet directional features for image semantic segmentation, particularly in the context of remotely sensed image analysis, have only been explored in a limited sense. This work aims to investigate the utility of directional features of contourlets in deep learning to identify informal settlements in remotely sensed images. The major contribution of this work is to propose a new model based on a set of multiscale contourlet masks as feature maps to include directional information in a deep learning framework with the help of approximation learning. Experimental results show that the proposed contourlet-assisted architecture is more effective than wavelet-assisted and plain networks in identifying informal settlements.

## 3. Essential Concept—Contourlet Based MRA

The central focus of this work is the utility of directional features of contourlet-based MRA in a deep learning framework. A machine learning approach basically learns various features that manifest different objects or classes in an image. The contourlet features have been used in different applications, including textural segmentation. The authors are motivated to utilize the directional features of the contourlet transform to assist a deep learning algorithm. 

The contourlet transform is implemented using a set of directional filters, which are designed using basis functions that have a choice of aspect ratios and directional orientations at multiple scales. In order to facilitate multiple scales, a Laplacian pyramid approach is combined with the directional filters [24]. The directional filter coefficients effectively capture the anisotropic relationship for curvilinear and disoriented edges. 

**Figure 1:**
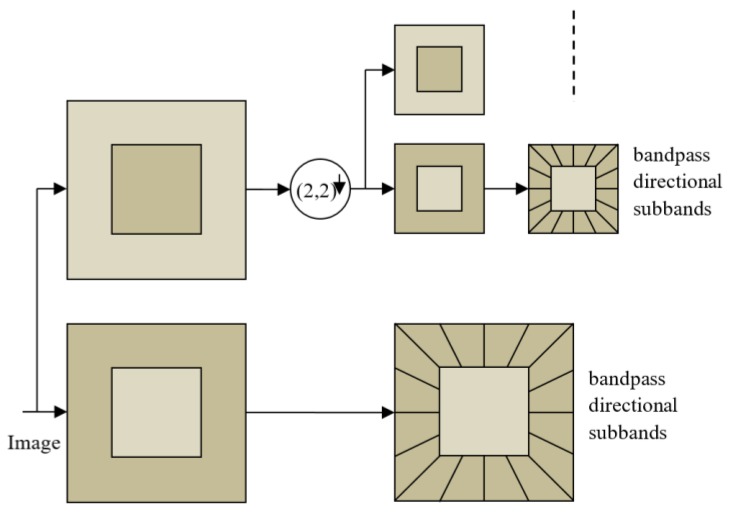
Contourlet transform decomposition structure (adapted from [24]).

The implementation of a contourlet transform facilitates any level of decomposition, a seamless transition from one scale to another, and faithful reconstruction. The numbers of directions and angular resolutions get doubled at every subsequent finer scale. Figure 1 shows a conceptual viewpoint of multiscale directional decomposition in terms of band-pass and low-pass filters and a down-sampler. The implementation details can be found in [24]. The low-pass filter outputs approximation level information, whereas the band-pass filter extracts the detailed information from a band. The process of decomposition can further be iterated in the low-pass filtered band to extract details of an approximation. These decomposed subbands are augmented with the layers of the U-net to provide multiscale learning, along with directional information. 

## 4. Proposed Method

In this paper, a modified version of the U-net [51] is used by augmenting contourlet masks of different sizes at different scales. During feature extraction, there are four steps, with the last three including several subbands. The feature maps in the same level have the same size, while the feature maps in the following level are half that of the previous level. For a 3-level decomposition, there are 16, 8, and 4 subbands respectively. The expansive part aims to extract feature maps for informal settlements using contourlet masks. The number of stages in contracting and expansive parts is the same. Having a convolutional layer followed by a max-pooling layer helps in gathering contextual information present at each level of decomposition in terms of generating activation functions. The decoder expands these activation functions with the help of the up-sampler and convolutional units to obtain the original size of a band. The central aim is to enhance the receptive field of the model using the down-sampler. The residual information in the process is fed to the up-sampler for faithful reconstruction. This is attained by the skip connections in the network, while the features learned during down-sampling are used in the up-sampling part. In turn, this mechanism provides smoother edges than other fully connected convolutional networks. 

**Figure 2:**
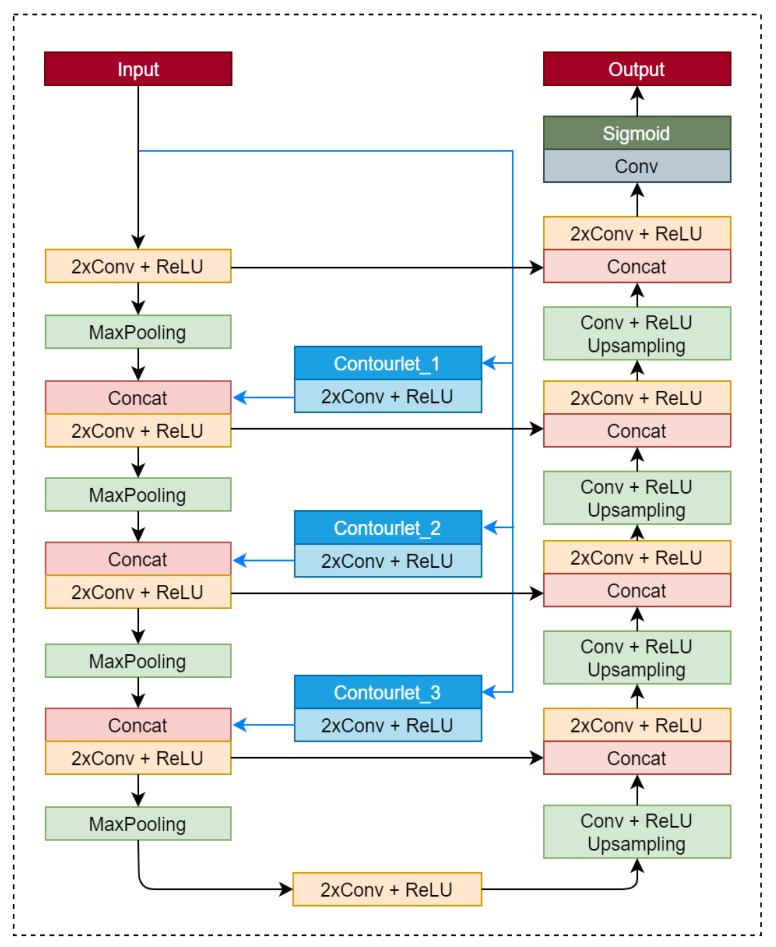
Proposed composite architecture (Conv: Convolution layer; ReLU: Rectified Linear Unit; MaxPooling: Maximum Pooling; Concat: Concatenation; Contourlet_x: Contourlet decomposed subband at level x)

Informal settlement identification is considered as a binary classification. For training, logistic regression is used by optimizing the energy function. A gradient decent algorithm is used to minimize the error function. Both the softmax and cross-entropy functions are considered for the error function. The softmax layer outputs two lines as a probability indicator for informal settlements and rest of the classes. The last layer is a convolutional layer measuring 1 x 1, which is used to transform the features into two classes for the pixel under consideration. The concatenation in the expansive segment is able to learn the features at multiple scales. The feature learning at multiple scales enhances the ability to capture different properties of the classes and improves the classification accuracy. The proposed architecture is shown in Figure 2.

## 5. Results

### 5.1. Dataset

The dataset comprises high-resolution Worldview-2 (2m×2m) images of parts of Mumbai and Pune cities from Western India. The study area is densely populated with a mixture of informal and formal built-up areas. Informal settlements in the region provide housing and livelihood for the lower economic strata population. In general, these informal settlements are very dense with clusters of row houses (called *chawls* in Mumbai). However, there are several discernible differences: areas that have been rehabilitated near high-rise colonies and towers; long-established localities, which have regular small-scale shops (called *kirana* in Mumbai) and roof structures alongside; highly congested pockets with only small lanes (parts of Govandi area in Mumbai); inner-city localities with high roof density (parts of PMG colony and Mankhurd) and small drainage lines (called *nallahs* in Mumbai).

### 5.2. Implementation and Results

The dataset comprises 1006 patches extracted from original images, including 878 patches for training, 38 patches for validation, and 90 patches for testing. These training patches were randomly sampled from the original images. For wavelet-based U-net, Daub (1, 2, 3, and 4) family and bi-orthogonal 9/7 basis functions were used to obtain the approximation and detailed subbands for three decomposition levels. The Adam optimizer with the second norm loss function was used in the training phase. The initial learning rate parameter was kept at 0.001 and decreased by a factor of one-tenth, with an epoch size of 20. 

In semantic segmentation, the low-pass filtered output is important, which approximates the input band while keeping the band-pass-filtered detailed information intact for better results [51,53]. With a limited number of samples, training a deep network might be very difficult. The authors in [53] used a pre-trained network to solve this problem. A quantum-inspired differential evolution method is used to fine tune the network parameters while training and to overcome premature convergence [54]. In order to achieve a reduced classification rule set, a particle swam optimization technique based on rough set theory is used to train a back-propagation network [55]. 

In order to have a pre-trained network, the authors used the approximation subband of Haar wavelet instead of original images for the training phase in the first step. After training on the approximation subband, the network weights were saved and a new U-net was created and trained on the original images. This was the second phase of training, where the network was initialized using saved weights from the first phase instead of random initialization. Here, the mapping from the original images to the original ground truth was learned. The expected result was that the network would able to learn the mapping faster than it would if it were to be trained on the original images with random weight initialization. By means of this transfer learning from the approximation subband, the network was able to learn the important features for segmentation at a given resolution; some of those features would also be useful for higher resolution original images.

The most common metrics used to evaluate a two-class classification method are precision and recall. The precision is calculated as the fraction of predicted informal settlement area pixels being labeled as informal settlements (IS), and recall is computed as the fraction of all labeled IS pixels that are correctly predicted. The precision and recall are also termed as correctness and completeness, respectively. F-score, mean intersection over union (mIoU), and overall accuracy (OA) are also used for quantitative assessment. With true positives (TP), false positives (FP), and false negatives (FN), the metrics are as follows:

- Correctness (C1) = TP/(TP + FP);
- Completeness (C2) = TP/(TP + FN);
- F-score = 2.C1.C2/(C1 + C2).

**Figure 3:**
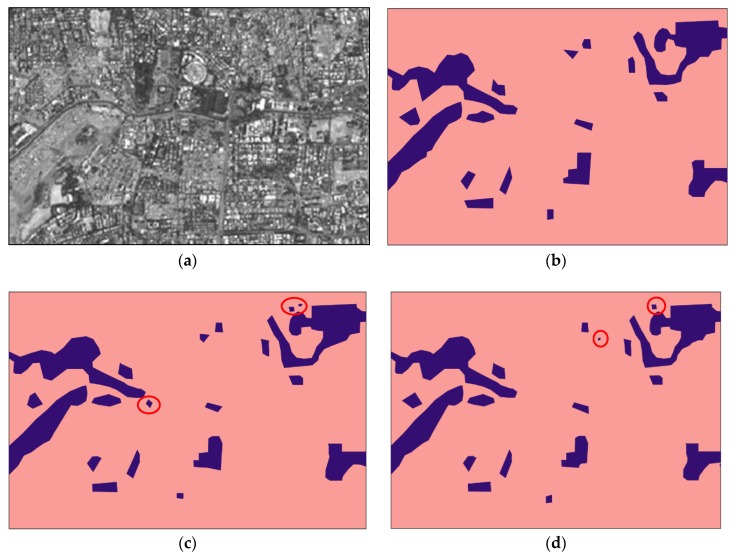

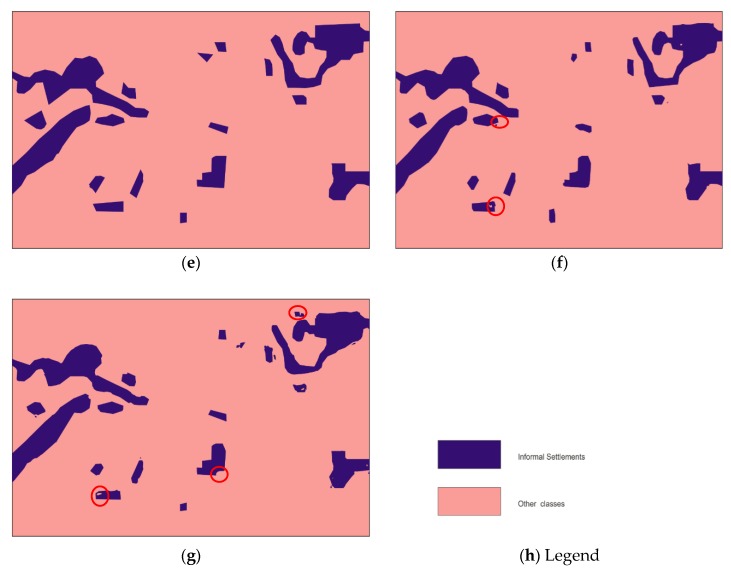
Informal settlement identification for parts of Pune city: (**a**) original image; (**b**) reference; (**c**) using plain U-net, with misclassifications circled in red; (**d**) using wavelet-assisted U-net, with misclassifications circled in red; (**e**) using contourlet-assisted U-net; (**f**) using contourlet texture features [20]; (**g**) using wavelet texture features [20]; (**h**) Legend.

**Figure 4:**
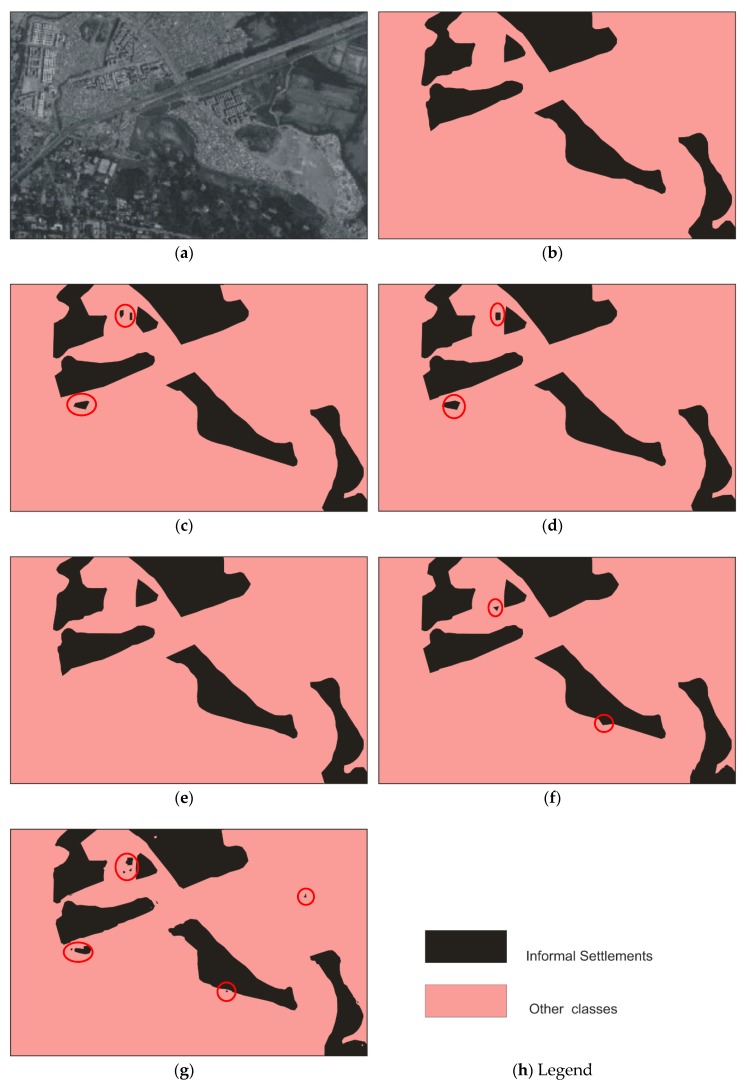
Informal settlement identification for parts of Mumbai city: (**a**) original image; (**b**) reference; (**c**) using plain U-net, with misclassifications circled in red; (**d**) using wavelet-assisted U-net, with misclassifications circled in red; (**e**) using contourlet-assisted U-net; (**f**) using contourlet texture features [20]; (**g**) using wavelet texture features [20]; (**h**) Legend.

Figure 3 and Figure 4 show the informal settlement identification results for Pune and Mumbai city images, respectively. Figure 3a,b show the original Pune city image and reference image, respectively. Figure 3c,d show the result using the U-net and wavelet-based U-net methods, in which it is observed that informal settlement areas were not identified properly and classes were misclassified in many locations. The misclassified portions are circled in red. The wavelet was found to be unsuitable for orientation and anisotropic properties of the classes, however the contourlet efficiently captured the curved boundaries and represented the disoriented and anisotropic properties of the structures. Figure 3e and Figure 4e demonstrate the utility of the contourlet features from all three decomposition levels, which show detailed directional information in the image. It is observed that informal settlements were correctly identified with better accuracy and improved edge continuity. The class does not appear to be intermixed with partially built-up or formally built-up areas, as observed while using the plain U-net method. This is due to the fact that the contourlet features capture the directional and isotropic properties of these classes. The accuracy in the boundary shape and edge continuity is observed to be better especially in the middle (reverse L-shape) and top-right potions of the image in Figure 3. Similarly, deviation from the reference image (Figure 4b) boundaries can be observed in the top-left and bottom-right portions of Figure 4c–e. Figure 3f,g and Figure 4f,g show the identification results using contourlet- and wavelet-based texture features [20]. The work in [20] emphasizes the utility of texture-based moment and energy features computed from MRA coefficients and does not utilize deep learning approaches for classification. It is observed that contourlet-assisted U-net performs better than the texture-based classification in terms of accuracy and visual interpretation. The misclassified pixels are highlighted with red circles in Figure 3 and Figure 4. 

The contourlet-assisted model performs better than the conventional U-net in terms of both pixel accuracy and mean intersection over union (mIoU). It is observed that Biorthogonal 9/7 is the best among all considered wavelet basis functions. However, the contourlet-assisted U-net outperforms all of the wavelet methods and the plain U-net method. The pixel accuracies and mIoU of the models for both the images using different methods are detailed in Table 1. As can be observed, the overall accuracy for Mumbai and Pune images improved by 3.04% and 3.76%, respectively. Additionally, precision and recall for informal settlements also improved compared to the results with plain and wavelet-based U-net methods.

## 6. Discussion

As described in the methodology, a comparative analysis was carried out by considering augmentation applied to the MRA features in deep learning.

1. U-cnet_1: Original Image + contourlet subbands at first level of decomposition;
2. U-cnet_2: Original Image + contourlet subbands at first and second levels of decomposition;
3. U-cnet_3: Original Image + contourlet subbands at first, second, and third levels of decomposition.

**Figure 5:**
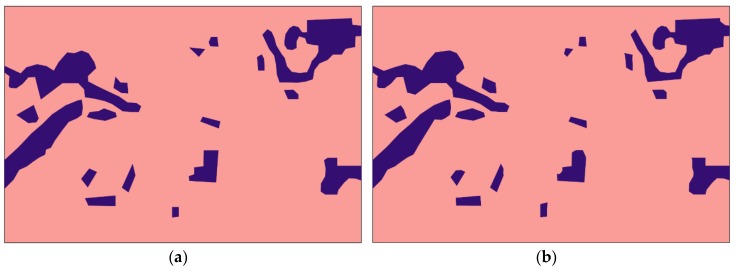

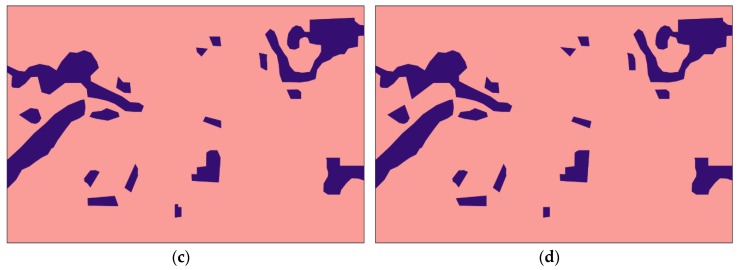
Effects of contourlet subbands for Pune image: (**a**) reference; (**b**) U-cnet_1; (**c**) U-cnet_2; (**d**) U-cnet_3.

**Figure 6:**
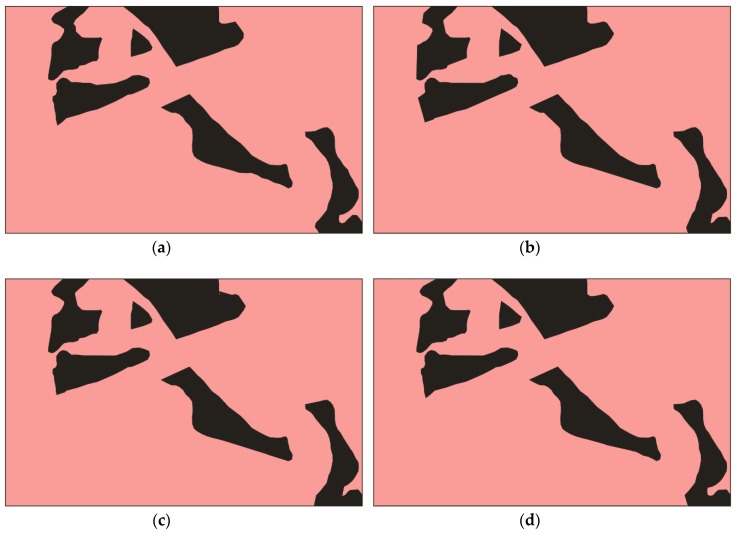
Effects of contourlet subbands with U-net for Mumbai image: (**a**) reference; (**b**) U-cnet_1; (**c**) U-cnet_2; (**d**) U-cnet_3.

The general trend was that the accuracies and mIoU for each image increased as the order of the decomposition increased. As observed in Figure 5 and Figure 6, U-cnet_3 (U-cnet of Figure 3 and Figure 4) outperforms other MRA methods, as it utilizes all 16 subbands containing directional details, which successfully capture the disoriented anisotropic features and irregular layouts of informal settlements. As observed from Figure 4, formal and informal settlements are mixed together in many places using U-net and wavelet-assisted U-net (U-wnet) models. This erroneous segmentation is due to the lack of finer details in plain U-net and wavelet-assisted U-net. This misclassification is also observed in U-cnet_1, as it does not incorporate all the subbands of the contourlet transform.

**Figure 7:**
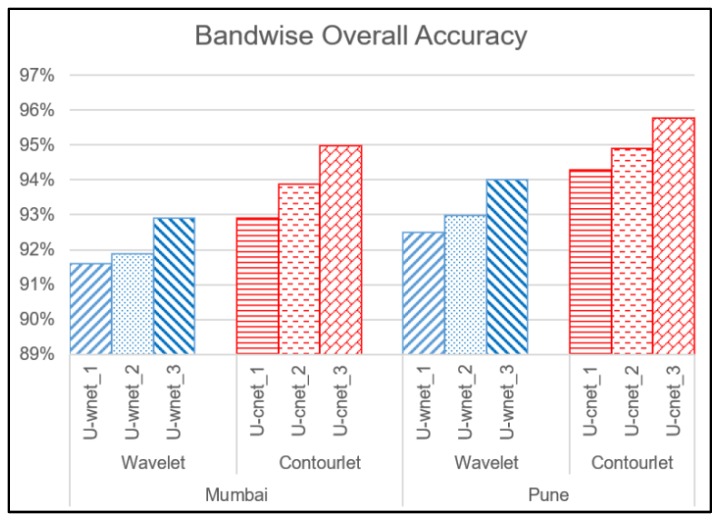
Band-wise overall accuracy.

**Figure 8:**
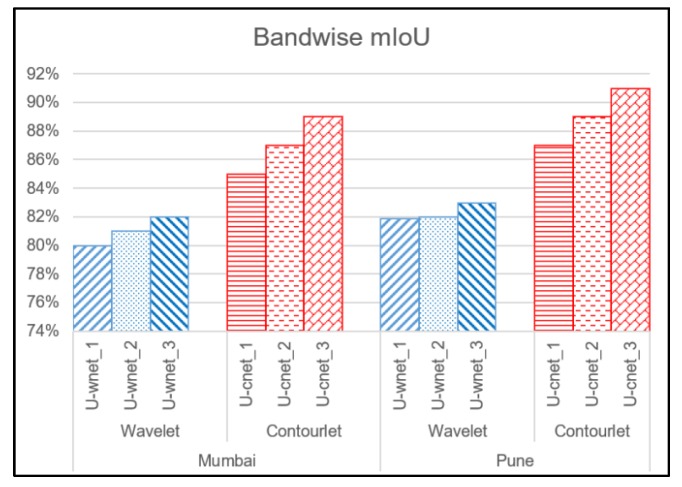
Band-wise mean intersection over union (mIoU).

Figure 5 and Figure 6 compare the band-wise results for the contourlet-assisted U-net. As the level of aggregation of subbands increases, the ability to capture intrinsic geometrical details and directional selectivity also increases, which in turn improves the identification accuracy of informal settlements. The U-cnet_3 model can analyze and recognise very small features of different classes containing rich and dense detailed information. This is also demonstrated using band-wise overall accuracy in Figure 7. Figure 8 presents mIoU with different levels of wavelet- and contourlet-decomposed subbands. For both the images, overall accuracy and mIoU using contourlet subbands is higher than that of wavelet subbands. Even the first level of the contourlet subbands (U-cnet_1) shows better performance than those of third level of wavelet subbands (U-wnet_3) for both of the images. The trend of overall accuracy is in agreement with the mIoU for the number of MRA subbands utilized in deep learning.

The results of a comparison with other methods using the same training and validation datasets are reported in Table 1. The proposed contourlet-assisted U-net method shows improvements in identifying informal settlements in both the datasets. The network extracts contourlet features at multiple scales, which works well in contraction, and it facilitates clear separation of disoriented boundaries.

## 7. Conclusions

In this paper, a deep learning method utilizing contourlet MRA features for identification of informal settlements using remote sensing data was proposed and analyzed. The major change proposed is augmentation of the directional subbands of the contourlet transform with a deep learning model. The method progressively combines subbands at various scales to extract different disoriented details, which are key manifestations of informal subregions in remotely sensed images. The proposed algorithm is tested on Worldview-2 images of Mumbai and Pune (India) covering different regions of formal and informal urban settlements. The results were compared with plain U-net and wavelet-assisted U-net models. The performance was evaluated based on the visual interpretation, precision, recall, F-score, mIoU, and overall accuracy of these methods.

The results showed that multiscale contourlet subbands in the proposed U-net yielded better class discrimination for both the datasets. The improved performance was because of the ability of the contourlet transform to capture directional features of linear and nonlinear discontinuities when compared with plain U-net and wavelet-assisted U-net models. The roof tops, boundaries of small lanes (*chawls*), irregular areas, and structures manifest as detailed edges in the image at different scales. These details are efficiently captured by the subbands of the contourlet transform, which show directional sensitivity and anisotropy. The contourlet subbands are capable of identifying the essence of informal areas—regions with particularly dense and jammed houses-which were not identified efficiently by the plain U-net model. The results for the contourlet-assisted model showed robust performance in terms of both visual interpretation and class identification, and are sufficiently robust against random pixels while preserving spatial regularity. An overall classification accuracy of 94.9–95.7% was attained with proper boundary shapes and edge continuity. The proposed model would provide local bodies a better mechanism to identify informal settlements in order to carry out advanced analysis in urban planning. The proposed method provides an option to local municipal corporations to enhance the efficiency of their often limited resources, especially due to prohibitive software licensing cost in developing countries, and to target support and improvement measures. Other non-wavelet-based MRA, such as curvelet and shearlet transforms, will be explored in a deep learning framework in future studies.

## Figures and Tables

**Table 1:** Performance comparison.

Model	Mumbai City Image					Pune City Image				
	C1	C2	FS	OA	mIoU	C1	C2	FS	OA	mIoU
U-net	0.9021	0.8812	0.8915	0.9194	0.79	0.9198	0.8878	0.9035	0.9202	0.81
U-wnet	0.9201	0.8902	0.9049	0.9290	0.82	0.9367	0.9018	0.9189	0.9401	0.83
U-cnet	0.9345	0.9101	0.9221	0.9498	0.89	0.9501	0.9198	0.9347	0.9578	0.91
WTex [20]	0.8135	0.8010	0.8072	0.8228	0.72	0.8247	0.8192	0.8219	0.8402	0.74
CTex [20]	0.9187	0.8992	0.9088	0.9201	0.82	0.9102	0.8994	0.9047	0.9224	0.81

U-wnet: wavelet-assisted U-net; U-cnet: contourlet-assisted U-net; WTex: wavelet-texture-based method; CTex: contourlet-texture-based method; C1: correctness; C2: completeness; FS: F-score; OA: overall accuracy; mIoU: mean intersection over union.
